# Psychosocial effects of adverse pregnancy outcomes and their influence on reporting pregnancy loss during surveys and surveillance: narratives from Uganda

**DOI:** 10.1186/s12889-023-16519-5

**Published:** 2023-08-18

**Authors:** Doris Kwesiga, Phillip Wanduru, Leif Eriksson, Mats Malqvist, Peter Waiswa, Hannah Blencowe

**Affiliations:** 1 Department of Health Policy, Planning and Management, School of Public Health, Makerere University, Kampala, Uganda; 2 Department of Women’s and Children’s Health, Uppsala University, Uppsala, Sweden; 3 Department of Global Public Health, Karolinska Institutet, Stockholm, Sweden; 4 Maternal, Adolescent, Reproductive & Child Health Centre (MARCH), London School of Hygiene & Tropical Medicine, London, UK

**Keywords:** Adverse pregnancy outcomes, Psychosocial, Surveys, Pregnancy loss

## Abstract

**Background:**

In 2021, Uganda had an estimated 25,855 stillbirths and 32,037 newborn deaths. Many Adverse Pregnancy Outcomes (APOs) go unreported despite causing profound grief and other mental health effects. This study explored psychosocial effects of APOs and their influence on reporting these events during surveys and surveillance settings in Uganda.

**Methods:**

A qualitative cross-sectional study was conducted in September 2021 in Iganga Mayuge health and demographic surveillance system site, eastern Uganda. Narratives were held with 44 women who had experienced an APO (miscarriage, stillbirth or neonatal death) and 7 men whose spouses had undergone the same. Respondents were purposively selected and the sample size premised on the need for diverse respondents. Reflexive thematic analysis was undertaken, supported by NVivo software.

**Results:**

60.8% of respondents had experienced neonatal deaths, 27.4% stillbirths, 11.8% miscarriages and almost half had multiple APOs. Theme one on psychosocial effects showed that both women and men suffered disbelief, depression, shame and thoughts of self-harm. In theme two on reactions to interviews, most respondents were reminded about their loss. Indeed, some women cried and a few requested termination of the interview. However, many said they eventually felt better, especially where interviewers comforted and advised them. In theme three about why people consent to such interviews, it was due to the respondents’ need for sensitization on causes of pregnancy loss and danger signs, plus the expectation that the interview would lead to improved health services. Theme four on suggestions for improving interviews highlighted respondents’ requests for a comforting and encouraging approach by interviewers.

**Conclusion:**

Psychosocial effects of APOs may influence respondents’ interest and ability to effectively engage in an interview. Findings suggest that a multi-pronged approach, including interviewer training in identifying and dealing responsively with grieving respondents, and meeting needs for health information and professional counselling could improve reporting of APOs in surveys and surveillance settings. More so, participants need to understand the purpose of the interview and have realistic expectations.

**Supplementary Information:**

The online version contains supplementary material available at 10.1186/s12889-023-16519-5.

## Background

Adverse Pregnancy Outcomes (APOs) including miscarriages, stillbirths and neonatal deaths remain numerous in Uganda, with the country’s stillbirth rate estimated at 15.1 per 1,000 total births in 2021 (based on 25,855 stillbirths), which is still high despite a 34.1% decline between 2000 and 2021 [1]. Uganda’s Neonatal Mortality Rate (NMR) in 2021 was estimated at 19 deaths per 1,000 live births (based on 32,037 newborn deaths). Many adverse pregnancy outcomes are preventable and survival of children is an important aspect of a thriving society.

Sustainable Development Goal (SDG) 3 on health has among its targets ending preventable deaths of newborns and children under 5 years, with newborn deaths to be as low as 12 per 1,000 live births by 2030 [2]. The Every Newborn Action Plan (ENAP) to end preventable deaths was launched in June 2014 [3] and it also set targets of 12 or fewer stillbirths per 1,000 total births in every country by 2030. Accurate data is needed for tracking progress in attaining the SDG and ENAP targets, assessing challenges, and is essential in understanding the scale of this public health problem plus planning for and financing interventions.

Surveys like the Demographic and Health Survey (DHS) are an important source of information on APOs in countries. They are also used for global estimates, for instance the United Nations Inter-agency Group for Child Mortality Estimation (UN-IGME) uses country DHS reports as part of source data for mortality estimates. Other surveys like Multiple Indicator Cluster Surveys contribute data for tracking outcomes, as do the Health and Demographic Surveillance System (HDSS) sites. However, surveys that collect stillbirth or neonatal deaths data have been noted to have issues with data quality, as does surveillance data, and require improvements [4, 5], without which underreporting of APOs will continue.

Furthermore, APOs have a big psychological impact on those who experience them. This includes an overwhelming grief that often lasts for several years [6]. Grief is a controversial concept, especially with regard to its intensity and duration [6, 7]. It includes the psychological, affective and physiological reactions after loss [7] and has also been defined as normal or complicated, with the latter reported as more severe [8]. Grief after an APO has many effects including depression, anger, self-blame and relationship breakdown [6, 7, 9, 10], maternal depression and other financial and economic costs to the family and community, many long term [11]. A review of literature on grief after miscarriage reported that while extreme grief declines and almost ends in six months, mourning continues [7] and grief is worse among women with repeated miscarriage [12]. A lot of this grief remains ignored [1].

These psychosocial effects may lead to failure to report the APO during surveys or surveillance, since disclosure could be affected by the respondent’s stage of grief. For instance a grieving woman may not want to resurrect memories of the loss that occurred, or does not see why she should share information that will not bring her baby back to life [13]. This will likely lead to under reporting of APOs, with some events not captured by interviewers.

This study explored the psychosocial effects of adverse pregnancy outcomes, so as to inform people designing and implementing surveys about possible challenges that may be encountered if respondents are grieving, which negatively influence data quality. It suggests how these can be handled to improve measurement of APOs. The study is based on the socio-ecological model [14], which posits that human behavior is influenced by multiple factors including individual, interpersonal, community, organizational and policy/enabling environment. It was deemed relevant because for instance psychosocial effects and their link to reporting of APOs are evident at the individual, interpersonal and community levels.

## Methods

### Study design

This was a qualitative study using an interpretative approach [15], undertaken in September 2021 in Iganga-Mayuge HDSS (IMHDSS). Narratives were conducted with women who had a neonatal death, miscarriage or stillbirth and men whose wives or partners suffered these events. This narrative approach involved interviews and was chosen because narratives provide respondents with a chance to mostly tell their own story, thus providing both the personal and the social, across a continuum of time (past, present and future) and place [16], some of which could be overlooked in a more structured tool.

This study was nested in the EN-INDEPTH study, a cross-sectional population based survey of 69,176 women of reproductive age, conducted between July 2017 and August 2018 in five HDSS sites (Bandim in Guinea Bissau, Dabat in Ethiopia, Iganga-Mayuge in Uganda, Matlab in Bangladesh and Kintampo in Ghana). The current study went in-depth in Iganga-Mayuge HDSS to further explore psychosocial effects of APOs, supplementing earlier work on barriers and enablers to reporting of pregnancy and adverse pregnancy outcomes in population based surveys [13].

### Study setting

Set up in 2004, IMHDSS, which is run by Makerere University Center for Health and Population Research is found in the two districts of Iganga and Mayuge, in east central Uganda, covering 65 villages within 7 sub-counties and 155 kms squared. Two thirds of the HDSS is rural (with 51% engaged in agriculture), and the rest peri-urban or urban. In 2017, IMHDSS had 94,568 people in 18,634 households, with 48% of the members below 15 years old. As an open cohort, IMHDSS collects data bi-annually on basic demographics including births and deaths, in addition to doing verbal and social autopsies [17]. Health facilities serving the area include 1 district referral hospital, 1 faith-based hospital, a total of 16 health center III and II, as well as Village Health Teams who mostly support health promotion. Iganga Mayuge HDSS was one of the sites of the EN-INDEPTH multi-country study that aimed to inform measurement improvements of pregnancy outcomes in population-based surveys.

### Participant selection

**Fig. 1:**
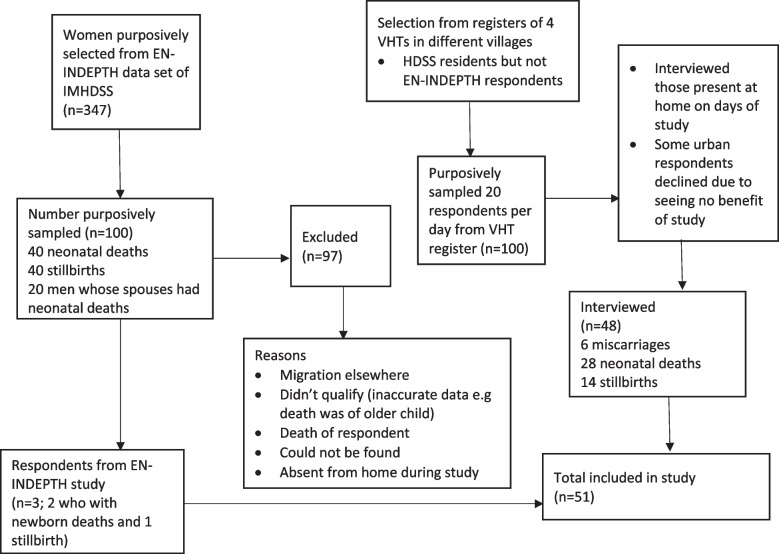
Respondent selection and sampling

Women and men were purposively selected from two sources (See Fig. 1). Our sample size determination was premised on the need for a diverse group of respondents, who may potentially have different experiences and perspectives, amidst the pragmatic needs of data collection in the available time [18]. We purposively sought to identify and represent a variety of women best placed to provide the information, across different APO categories, ages (15–49 years old) and urban and rural settings, as well as men whose wives were known to have suffered an APO and who were willing to participate in the study.

### Data collection

Data were collected using a narrative guide that had three questions: one on the respondent’s experience during the APO (interviewers let the respondent narrate her/his story here and probed where clarity was needed); another on whether they were able to talk to people about the loss, especially to interviewers in the past if they were previously interviewed and in the current study, and finally we asked for suggestions on how to improve data collection on APOs during surveys.

Most interviews were held at the home of the respondents, with one held at the local health center. Privacy was assured by conducting the interview with the respondent away from other people. All interviews were conducted in *lusoga* and audio recorded on tablets or mobile phones. Each interviewer then transcribed their own set of interviews into English. Transcription started in the field alongside data collection and was completed within one month of data collection ending. At the end of each day, the field team discussed the activity and observations, which the main author wrote as field notes that supplemented the analysis.

Each respondent provided written informed consent. The EN-INDEPTH study received ethical approval from various country institutional review boards and Uganda National Council for Science and Technology (UNCST) while the PhD study also received ethical approval from Makerere University School of Public Health in Uganda and UNCST.

Additionally, due to the sensitivity of the study topic, a lot of effort was put into teaching the data collectors during pre-field training about interviewing grieving respondents. We followed a protocol developed for the earlier EN-INDEPTH study phases that showed the interviewer what to do if the respondent was distressed, for instance pausing the interview and if able, resuming it later. Interviewers inevitably encountered these situations and used the pauses to verbally comfort the respondents or changed the topic briefly. They also used a gentle probing approach where respondents were giving brief answers due to their emotional state. In most cases the respondents were able to resume the interview. However, some were too distraught to talk and the interviewers had to end these sessions prematurely.

We used narratives to let the respondents speak more freely but also kept the questions to a minimum and hopefully in this way reduced social desirability bias that would have come up through multiple questions, some of which could be leading or embarrassing. The interviewers did probe but they were well trained to ensure a sensitive and non-judgmental approach, focusing on the experiences shared.

### Data analysis

Reflexive thematic analysis was undertaken in six steps as recommended by Braun and Clarke: familiarisation; coding; generating initial themes; reviewing and developing themes; refining, defining and naming themes; and writing up [19]. Familiarization involved reading through the transcripts and taking down brief descriptions of potential codes and meanings. This was followed by inductive coding using NVivo software (version 12) that involved reading each transcript and identifying codes. NVivo was also used for generation of coding reports, including the coding summary by code report and the code summary report.

Coding was done by DK and PWd, who first coded separately and then had physical meetings to discuss each code developed and decide what to merge or discard. Initial themes were generated from the codes by grouping similar codes together and getting into an iterative process of regrouping the themes alongside further review of the codes under each and their meanings. Theme development was done by DK, with HB joining in subsequent discussions about themes. Other authors also helped to further refine the themes after the first draft of the paper was written. Respondents’ own words were used for the titles of themes to try and provide for the reader as realistic or authentic as possible a chance to understand the psychosocial state of the respondents and their desires, without having been present in the field. Attention was paid to identifying the similarities and differences between the women’s and men’s experiences as well as across the various APOs. We chose to analyze all 51 interviews so as to not miss out or any important data across the different respondent categories.

### Research team and reflexivity

The narratives were conducted by a team of five research assistants (WC; DM; JI; BK and SN) who have long term experience in conducting qualitative studies not only in Uganda but also within IMHDSS. The lead author, DK, attended some interviews and was present in the field throughout the data collection. Analysis and writing were led by DK, supported by the other co-authors. All authors are working in the public health field and acknowledge having prior knowledge of psychosocial effects of pregnancy loss. This played a role in conceptualization of the study and in the analysis process, since we were already aware of some contextual issues.

### Trustworthiness of findings

This is demonstrated through the concepts of credibility, transferability, dependability and confirmability in the following ways: Credibility was enhanced through data triangulation and investigator triangulation [20]. Dependability of findings is illustrated through the well documented methods and the reflexivity section. Triangulation and reflexivity are also indicators of confirmability of the study findings [21]. Study findings are derived from the data and indeed if a confirmability audit were to be done, the results could be verified.

## Results

We interviewed 51 respondents, majority of whom were females between 20 and 39 years and who had experienced neonatal deaths (Table 1). Half of them engaged in subsistence farming as a source of income. Interview length was an average of 40 min, with the shortest being 27 min and the longest 1 h and 14 min.

**Table 1:** Demographics of the respondents

Characteristic	Number (%)
**Sex**	
Females	44 (86.2)
Males	7 (13.7)
**Age**	
15–19	4 (7.8)
20–24	17 (33.3)
25–29	9 (17.6)
30–34	9 (17.6)
35–39	8 (15.6)
40–44	3 (5.8)
45–49	0 (0)
50–55	1 (1.9)
**Education level**	
None	1 (1.9)
Lower primary (P.1-P.3)	5 (9.8)
Upper primary (P.4-P.7)	24 (47)
O-level	17 (33.3)
A level	2 (3.9)
University	1 (1.9)
*Missing*	*1 (1.9)*
**Most recent adverse pregnancy outcome**	
Miscarriage	6 (11.7)
Neonatal death	31 (60.7)
Stillbirth	14 (27.4)
**Number of adverse pregnancy outcomes per person**	
1	27 (52.9)
2	17 (33.3)
3	7 (13.7)
**Total**	**51**

### THEMES

We present our results under four themes: 1) I cried a lot and I still cry (highlights the psychosocial effects of the APO on respondents); 2) It is hurting me afresh (people’s reactions to interviews about their loss); 3) You never know, the person can give you some advice (why people agree to interviews about APOs); and 4) You are supposed to give us counseling and we come back to normal (respondents suggestions for improving interviews on APOs).

### I cried a lot and I still cry: psychosocial effects of the APO on respondents

In this section, we highlight the various psychosocial effects reported during this study, most of which were negative.

**Fig. 2:**
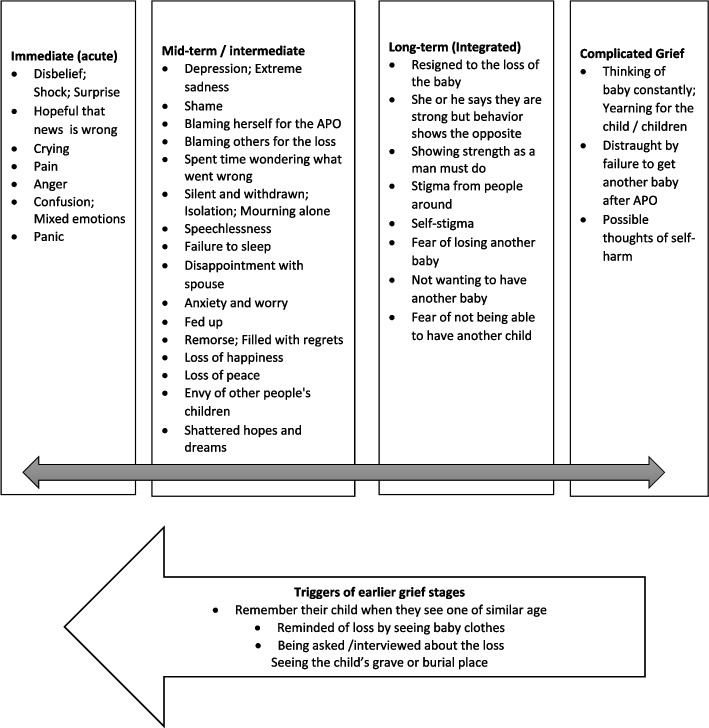
Psychosocial effects of adverse pregnancy outcomes

In Fig. 2, we grouped the psychosocial effects in four broad categories, recognizing that grieving is a journey that progresses in phases although it is also cyclical, with a possible return to an earlier stage depending on the triggers. We adapted the categorization by Shear and others, who defined the initial response as acute grief, followed by integrated grief where the bereaved resumes their daily life and complicated grief that is prolonged and may be considered a mental health disorder [22].

These effects manifested regardless of the type of pregnancy loss – whether one had experienced a miscarriage, stillbirth or neonatal death. The difference was that respondents who had suffered multiple APOs appeared to face more intense and longer lasting psychosocial effects, as did those who lost their first baby or who did not have any other living children. In instances where women already had other living children, they were comforted to some extent by these, as well as by other children they delivered after the APO. Where a woman had tried to have another baby after the APO but failed, her stress increased.

*It hurts me a lot. I was depressed and I even told my husband that I am leaving, going back to my parent’s home. Even the things he had bought for his baby I told him that let me take them and give them away because whenever I would look at them, I would feel sadness, but he said no. I cried a lot, I started crying when the water broke and even when they were removing the thing, I was just crying but the midwife said be strong and I save your life (Woman after miscarriage)*

Due to the negative psychosocial effects, many respondents suffered physical effects, including fainting on hearing about the death, readmission to the health facility, falling sick and failing to do their chores (for women, some had prolonged sickness after birth), and a few men also had their physical health suffer. Many reported loss of physical energy and appetite.

Of the seven men we spoke to, most revealed that they did suffer the pain of loss, including depression and anxiety, crying and disbelief. One man described how he fell sick and had to quit work after the APO. Many of them explained that their wives were more distraught and the men had to be strong and comfort them instead. A major worry that the men had was the financial losses they incurred due to high expenditure when the pregnancy was also problematic, while the APO occurred and after. Some mentioned borrowing money and losing out on work during that time, which caused them a lot of psychosocial stress.

*Even up to now I am sad because sometimes, there are certain things that you buy for the child, but whenever you look at them, you feel sad. Whenever you pass near the grave and look there, you feel sad. When you look at someone with their little child, you remember yours, yes (Man after neonatal death)*

In the majority of cases, respondents did not know what had caused the death of their baby. They reported that health workers barely explained what happened and often they appeared not to even acknowledge the emotions around the loss. This lack of information, poor communication and perceived hostility from health workers increased confusion and distress around the APO, with respondents unsure what to do to avoid future pregnancy loss. We also found a few cases of gender based violence towards women by their husbands/spouses, both during and after the pregnancy, increasing the psychosocial problems, recovery and general unhappiness.

### It is hurting me afresh: people’s reactions to interviews about their loss

Here we specifically referred to the respondents’ reactions to being interviewed about the adverse pregnancy outcome in the current study, as well as during previous interviews, including the EN-INDEPTH study and HDSS interviews. Although responses varied, over three quarters of respondents, both women and men, acknowledged that the interview(s) reminded them about their loss and felt like they were reliving the event. This led to a feeling of deep sorrow and crying in a few instances. While less than a quarter said they had recovered from the loss, they still pointed out that things or events come up that remind them about it and drag them backwards, so they preferred not to discuss it.

It was frequently explained that discussing the loss of the baby was difficult and disheartening and a few people in this study requested the interviewer to change the topic, while a handful requested for that part of the interview to be stopped. Some expressed their desire to discuss other things except the APO. Indeed, we had a number of cases where respondents, mostly women, broke down and cried during the interview and the interviewers had to give them time to compose themselves and continue the discussion where able, or stopped the interview. Numerous respondents stated that such interviews left them feeling depressed but explained that later on they would pick themselves up. A few others mentioned feeling sad but with no choice except to engage in the interview, while a handful were very silent and the interviewers struggled to get responses from them. When asked what they felt on being interviewed, they kept quiet.

*…Talking about it that Mrs so and so got a stillbirth…I would not want to hear it. I wanted that maybe if we are seated discussing they should not include that, we discuss other things (Woman after a stillbirth)*

On the other hand, about a quarter said they were okay with interviews and did not have any negative feelings and that the loss was in the past. However, they also linked this to the need to be strong and avoid returning to depression and worry. In fact, when asked if they wanted counselling, a minority of respondents declined, stating that they had already recovered.

Interestingly, many respondents shared that at the end of the interview, they eventually felt better, especially where interviewers comforted them, advised, strengthened and encouraged them and it was an opportunity for letting off steam. In fact, one woman described it as “mourning with her”, thus rejuvenating her. A few others compared it to receiving visitors or friends who cheered you up or in whom you confided, which was better than suffering with the pain alone. Another explained that her happiness from the interview was due to realizing that there were people concerned about pregnancy loss and interested in solving the problem.

*It’s like when you have a problem and confide in your friend, you feel better. It can’t be compared to keeping something to yourself. The pain is too much and you feel it alone (Woman after neonatal death)*

Nevertheless, it was evident that recovery from the loss is a slow and gradual process, with lots of back and forth emotions, where somebody may be strong for a while and then something happens that reminds them of the APO. Indeed, while some people affirmed being strong and having moved on from the past, responses showed that you can never totally move on or forget the loss.

### You never know, the person can give you some advice: why people agree to interviews about APOs

Among key issues identified was that when somebody consents to an interview about an APO, they also have certain expectations from the interviewers to whom they open up or the people going to receive the information. This influences whether they agree to participate and how open they shall be with their answers. We found that respondents often agreed to interviews because they wanted advice on how to recognize danger signs during pregnancy or after giving birth and what to do, so that the next time they could rush and seek appropriate care for the mother or baby and thus save lives. Generally, almost all respondents in this study indicated that they wanted sensitization on understanding the cause of pregnancy loss and what to do to prevent the reoccurrence of the APO and hoped to get this advice or other help from the interviewer or the study.

Furthermore, about three quarters of respondents wanted tangible, visible and useful benefits from the interview, not just reporting and sharing. They wondered whether reporting the APOs was going to result in medicines brought to the health centers, a better attitude among health workers and improved service delivery in health facilities for mothers and babies, free treatment in health centers, treating the diseases in the body that cause pregnancy loss and more. This was partly because some linked their loss to poor quality of care in the health facilities so they hoped for improvement. One was explicit about the need to take information back to the government about the inequitable treatment by health workers. Indeed, many were keen to know the outcome of the interviews and directly asked about this. Additionally, some people hoped to receive advice from the interviewer about how to handle or avoid the depression that comes with that kind of loss.

*I had expected that he would give me advice on what to do but he just recorded his things and left. I felt bad (Woman after miscarriage)*

*I felt happy sharing with you my experience and confiding in you because you can advise me (Woman after stillbirth)*

*After telling you, what advice do you give me so that I don’t get depressed? So I ask you to advise me and you tell me to do this…. It is you to teach me how to stop the depression (Man after neonatal death)*

### You are supposed to give us counseling and we come back to normal: respondents’ suggestions for improving interviews

We asked respondents to suggest ways in which interviews about APOs can be improved, so as to lessen the pain they cause to the bereaved. We broadly categorize these into content and approach/process.

In terms of content, over half the respondents wanted to be asked about how they were treated while at the health facility, the response of the health workers and other patient care questions. They emphasized that these were important especially since some of them had complaints about the health workers and how the process of their loss was handled. Furthermore, some wanted to be asked what exactly happened to cause the death and therefore receive advice as earlier mentioned. These questions around occurrence of death, especially for those who went to the facility, were often recommended.

With regard to the process, respondents appreciated interviewers who comforted, encouraged and strengthened them while they spoke about the loss, exhibiting empathy and concern. The need for a polite and calm manner was requested for and there were a few suggestions to bring condolences like the HDSS does, financial incentives, or in-kind gifts like food.

There were a few suggestions that professional counselling and sensitization be included alongside the interview. Indeed when we asked each respondent whether they would be interested in receiving professional counselling after the study, more than three quarters responded positively. Only a minority did not want counselling, expressing the fact that they had moved on from the loss already. However, on deeper analysis we realized that people mostly wanted education, to know what went wrong, why and how to avoid it in the future, rather than traditional counselling approaches used for instance in psychology.

*It needs counseling and sensitizing that since she faced that loss, it’s better she always visits health professionals in case of any danger signs and you give her such advice so that by the time she gets pregnant, she would be already informed (Man after neonatal death)*

The length of time after which interviews could be done was only spoken about by one person, who asked that interviews are conducted only when someone has accepted the loss. At a more general level, a few respondents pointed out how the community entry and household strategies could be improved, through starting with the local councilors (*LC1* is a local community leader in Uganda), so they could notify the households about the study. This advance notice would give respondents time to prepare and avoid abrupt visits from interviewers.

One respondent mentioned the benefit of the narrative approach that we used in this study, because we let the respondent tell their story and she found this easier than the frequent question and answer approach. Overall, however, most respondents appreciated that the researchers had to do their job amidst a difficult situation.

*Like the way you have come to ask me, it’s good to first go through the LC1 of our area and that person mobilizes us and informs us that we will receive visitors. It’s good to inform someone in advance that I am going to visit you and this is the purpose for my visit. But there is someone just coming abruptly to someone’s home… (Woman after neonatal death)*

## Discussion

It is known that after occurrence of an adverse pregnancy outcome, both women and men suffer psychosocial effects, including complicated grief, depression and more. Many studies have been conducted in high income settings [23–26], with much less published in low income settings [27–29]. Furthermore, reluctance to talk about APOs has been reported, including during interviews, potentially affecting the quality of data, although the link between APOs and reporting during surveys and surveillance settings is much less written about [13].

This study shows the range of psychosocial effects suffered by both men and women after an APO, presented as acute, intermediate, long term and complicated grief. These occur regardless of APO type. Our outcomes were similar to other studies, where APOs have been reported to cause extreme grief, crying, men wanting to appear strong, anxiety about the next pregnancy, envy, anger, a feeling of shame and guilt and others [11, 12, 28, 30]. The study further shows men being more prone to grief suppression so as to support the mother of the baby, as has been noted elsewhere [23, 30]. Additionally, men were often anxious about the financial aspect of the APO.

Furthermore, this paper describes people’s reactions to interviews about the APO. In most cases, they shared that interviews revived bad memories. While conducting research on sensitive topics like pregnancy loss, it is important to understand that the respondent is grieving and this grief does not necessarily end, regardless of how far back the event happened. One example is Verbal and Social Autopsy (VASA), a method employed to determine the cause of death by asking family members about the events preceding the death [31, 32]. VASA has also been associated with emotional distress on the side of both respondents and interviewers, especially among parents [33, 34].

However, this study also showed the potential therapeutic effects of a well conducted interview, when respondents shared that sometimes they feel better after the interview, having received encouragement and advice. In Papua New Guinea, respondents reported being comforted and strengthened by the verbal autopsy interview, even amidst sadness [34].

This paper was unique in its interrogation of why people agree to interviews about APOs. Respondents’ expectations included sensitization on pregnancy loss. More so, people agree to the interview because they expect tangible benefits not just at individual level but society and community as well, for instance better health care. People appear to be seeking for help in a community where there is none. This study also adds knowledge of community members’ suggestions for improving sensitive interviews. This includes asking about the birth process but also the need for interviewers to be polite, calm and offer counselling. More so, we identified confusion between the terms “counselling” and “health education” in the study setting. While the research team offered counselling, as per the one-on-one psychology approach, it transpired that what respondents understood as counselling was actually health education.

The strength of our study was the large number of narratives conducted with people who had different types of APOs. The use of the narrative approach was also beneficial because the respondent was able to talk more easily and share information that we likely would have not asked about in a more structured guide. The study was limited by being done in one geographical area so elsewhere some findings may be different. However, the fact that psychosocial effects are similar to those in high income settings indicates differences may be few. What may differ, perhaps, is their expectations from interviews.

This study has various implications for interview practice both now and in the future, within the context of surveys, surveillance and beyond. We thus provide suggestions for improving interviews involving questions on APOs during surveys or routine surveillance.

### Interviewer training on identifying and handling grief

Interviewers need training in dealing with grieving respondents, including awareness of reactions to expect, like crying or silence that are sometimes mistaken for one simply not wanting to participate. Events surrounding pregnancy loss are complex [32] and interviewers must be adequately trained to collect accurate and complete data. For instance elsewhere, contextually appropriate interviewer training in bereavement counselling has been recommended for verbal autopsy, to ensure interviewers can handle the distress likely to occur [35].

Furthermore, interviewers need specialized training [36] around the ethics of conducting such interviews and should also know where to refer the extremely troubled respondents. In a previous study, interviewers reported pretending to also have had an APO, so as to get respondents to confide in them [13]. While they saw this as empathy, it may be unethical, hence the need for training on ethics.

### Provision of health education

In relation to people’s expectations from interviews on pregnancy loss, it is imperative that interviews on APOs be conducted with a component of health education, where respondents are concurrently provided with information on what causes the various kinds of pregnancy loss and how these could potentially be avoided in the future. This could be done in two ways; firstly by incorporating this information in the interviewers’ training, so they are able to explain it verbally to the respondents. Another option would be to provide printed, simplified information in the predominant local language that an interviewer could leave with those who have had APOs, because in a survey context like the DHS, time for in-depth discussions is limited.

### Incorporating counselling or referrals for counselling

More so, there is a need to link the grieving parties to counselling services where available. This could again be through interviewers sharing the relevant contact information, or study organisers embedding this into their research, with a trained counsellor accompanying the interviewers, not only as a component of giving back to their respondents but also as an ethical approach to data collection among grieving people as has been recommended in the ethical principal of justice [37]. It is important to provide some benefits to respondents, not just burdens [33]. In Australia, interviewers in a study with people who lost children undertook a course on bereavement counselling, in addition to the researchers referring some respondents to a social worker where necessary [38].

However, in view of limited existence of static counselling services in the study setting, especially in rural areas, surveys could approach individual psychologists, social workers or counselors to work with for the length of the study. Indeed, Gouda and others recommended collaboration with local psychologists during verbal autopsy studies [35]. They also recommended grief-sensitive research protocols. Indeed, in a study in Kenya, interviewers felt they could not adequately comfort the bereaved during verbal autopsy interviews due to inadequate counselling skills and wanted formal counselling training [33]. However, flexibility around time may also be a barrier for interviewers, even when trained in grief counselling.

### Respondents’ understanding of the interview purpose

It is critical that from the outset, respondents clearly understand the purpose of the interview and the role of the interviewer. This will ensure that they have realistic expectations and are not disappointed or resentful after sharing information. This can be done in two ways: firstly, through a good community entry and engagement process before the initiation of the study. If the local leaders and other key opinion leaders are informed about the study and are instrumental in mobilizing respondents, in addition to communication over appropriate media, then respondents will be more prepared [39]. Secondly, a clear consenting process at the start of the interview is key.

All the above recommendations are also relevant for the HDSS sites which conduct regular surveillance on various indicators including APOs. For instance, although the bulk of the Verbal and Social Autosy tool is quantitative, it has an open ended section for the respondent to narrate what happened to the deceased. The quality of this narrative has been noted as dependent on the interviewer’s training and skill, with one study showing discrepancies in the open narrative section when two interviewers concurrently recorded an interview [40]. While some of the recommendations may be hard to implement within surveys like the DHS due to its nature, we believe lessons can be applied to other research studies. This is particularly important for research on sensitive issues where substantial underreporting has been reported during standard surveys, for instance on sexual and gender based violence [41].

### Future research

Interviews integrating bereavement counselling and /or health education are needed to see the effect on reporting of APOs and on the resultant psychosocial state of grieving respondents and quality of data shared.

## Conclusion

Psychosocial effects of APOs may influence respondents’ interest and ability to effectively engage in an interview. Findings from this study suggest that a multi-pronged approach, including interviewer training in identifying and dealing responsively with grieving respondents, and meeting needs for health information and professional counselling could improve reporting of APOs in surveys and surveillance settings. More so, participants need to understand the purpose of the interview and have realistic expectations.

### Supplementary Information

**Additional file 1. **Narrative guide. This was the interview tool used.

**Additional file 2.** Ethical approval for EN-INDEPTH study from local institutional review boards. Shows the dates when ethical approval was received in each site and the reference number.

## Data Availability

The datasets (transcripts of narratives) generated and analysed during the current study are not publicly available due to the need to protect privacy of participants but are available from the corresponding author on reasonable request.

## Abbreviations

APOs: Adverse Pregnancy Outcomes
DHS: Demographic and Health Survey
ENAP: Every Newborn Action Plan
EN-INDEPTH: Every Newborn-International Network for the Demographic Evaluation of Populations and their Health
HDSS: Health and Demographic Surveillance System Site
IMHDSS: Iganga Mayuge Health and Demographic Surveillance System Site
LC: Local Councillor
NMR: Neonatal Mortality Rate
SDGs: Sustainable Development Goals
UNCST: Uganda National Council for Science and Technology
UN-IGME: United Nations Inter-agency Group for Child Mortality Estimation
VASA: Verbal And Social Autopsy
