# Supplementary material for: Psychosocial effects of adverse pregnancy outcomes and their influence on reporting pregnancy loss during surveys and surveillance: narratives from Uganda
Source: BMC Public Health. 2023 Aug 18;23:1581. doi: 10.1186/s12889-023-16519-5 (PMC10439567; doi:10.1186/s12889-023-16519-5)
Supplement: Supplementary file 1 — Additional file 1. Narrative guide. This was the interview tool used. [file 12889_2023_16519_MOESM1_ESM.pdf]

Additional file 1: Narrative guide

(Women who have had a neonatal death, miscarriage or stillbirth; Men whose wives / partners have had these events)

Interviewer ID ___ ___ ___ ___

Respondent ID ___ ___ ___ ___

Interview date: __ __ / __ __ / ___ ___
(*DD/MM/YY*)

Informant HDSS ID	Age:	Marital Status:
Location of Interview:	Religion:	Occupation:
Years of Education:	No. of living Children :	No of pregnancies lost **(ask about this later, not at the start)**<br><br>Type of adverse outcome

Introduction:

- Introduce yourself
- Ensure the informant is comfortable, has time, and is able to participate in the interview.
- Provide information about the study. Review the purpose of the interview with the informant.
- Explain to the informant what to expect of the interview.
- Explain to the informant that:
  - You especially want to learn about her experiences and thoughts on this topic
  - There is NO 'right' or 'wrong' answer.
  - Her answers will be put together with the answers of other people to help get a wide understanding of what people think about this topic.

Then read the consent form and obtain the informant's consent to proceed with the interview. DO NOT proceed without informed consent. Start the audio recorder.

Time interview started: ☐☐:☐☐

Instructions to the interviewer: *This is a narrative and focus is on the respondent explaining the situation that they went through. That is why the tool has very few questions. Once you ask a question, please allow the respondent to explain the experience in their own words. Probe occasionally and keep prompting for more information until the respondent says she has nothing else to add.*

Part A: Experience with adverse pregnancy outcomes (neonatal deaths, stillbirths, miscarriages, abortions)

Sometimes, as you know, a woman may be pregnant but unfortunately the baby dies. The baby may die in the first months of the pregnancy, later on after seven months, or while she is giving birth. The baby can also be born alive, but dies before one month has passed. I am talking to you because you had this unfortunate experience. I now want to talk about these deaths, and what happened when you lost your baby. It is a sad topic, but it will later help us to know why the babies die and what we can do to save other babies lives.

1. Please tell me about the pregnancy loss(es) that you suffered. How did it happen? What was your experience with the loss?
   Probe: What did you do? How did the death affect you? (*things like depression, sadness, crying, failing to do your work*)
   Probe: How did you manage to cope or deal with this loss? Have you felt like you are getting back to how you were before?

2. Were you able to talk about the loss? If yes, whom did you tell?
   Probe: Barriers faced in telling people about your loss; what made it easier? What made it harder?
   Probe: **For the women**: What about when interviewers came to ask you about it? (*explain that this was in 2018, when a few women in the HDSS were interviewed?*)
   How did you feel? What did you do? Did you tell them what happened? Did you feel better or worse after sharing with them?
   Probe: For the men: Has any interviewer ever asked them about this loss? If yes, how did they feel when asked? What did they do? Did you tell them what happened?

Part B: Recommendations

Finally, we would like to conclude our discussion by asking for your advice.

3. What do you think can be done so that when interviewers come to talk to people who have lost babies, they can get better information without causing so much pain to the mother?
   Probe: What kind of questions could they ask? How could they ask them?

Offering counselling

Now Sir/Madam, we would like to offer you counselling to help deal with your loss. Have you spoken to any counsellor about your loss? Do you mind talking to somebody who has been trained to do this? It will not cost you any money. The person will just listen to you and offer you some comfort and advice.

(If respondent agrees, please list their names and share with Doris right after interview)
