# Supplementary material for: Psychosocial effects of adverse pregnancy outcomes and their influence on reporting pregnancy loss during surveys and surveillance: narratives from Uganda
Source: BMC Public Health. 2023 Aug 18;23:1581. doi: 10.1186/s12889-023-16519-5 (PMC10439567; doi:10.1186/s12889-023-16519-5)
Supplement: Supplementary file 2 — Additional file 2. Ethical approval for EN-INDEPTH study from local institutional review boards. Shows the dates when ethical approval was received in each site and the reference number. [file 12889_2023_16519_MOESM2_ESM.pdf]

Additional File 2: Ethical approval for EN-INDEPTH study from local institutional review boards

Site	Institutional Review Boards	Date	Number/Ref
Bandim (Guinea-Bissau)	Comité Nacional de Ética na Saúde	12 June 2017	072/CNES/INASA/2017
Dabat (Ethiopia)	Institutional Review Board, University of Gondar	19 April 2017	VP/RCS/05/1074/2016
IgangaMayuge (Uganda)	Mildmay Uganda Research Ethics Committee<br><br>Uganda National Council of Science and Technology	26 June 2017<br><br>11 October 2017	REC REF 0305-2017<br><br>SS 4244
Kintampo (Ghana)	Kintampo Health Research Centre, Ghana Health service Scientific Review Committee (SRC)<br><br>Ghana Health Services Ethics Review Committee<br><br>Kintampo Health Research Centre Institutional Ethics Committee	14 June 2017<br><br>26 July 2017<br><br>9 August 2017	SRC/130617<br><br>GHS-ERC:19/06/14<br><br>KHRCIEC/2017-14

Matlab (Bangladesh)	Icddr,b Ethical Review Committee	19 July 2017	PR-17049
London School of Hygiene & Tropical Medicine (United Kingdom)	London School of Hygiene & Tropical Medicine	24 May 2017	12218
